# Preparation, optimization of the inclusion complex of glaucocalyxin A with sulfobutylether-β-cyclodextrin and antitumor study

**DOI:** 10.1080/10717544.2019.1568623

**Published:** 2019-03-21

**Authors:** Lili Ren, Jingjing Wang, Guoguang Chen

**Affiliations:** a School of Pharmacy, Nanjing Tech University, Nanjing, China;; b Department of Microbiology and Immunology, Stanford University, Palo Alto, CA, USA

**Keywords:** Glaucocalyxin A, SBE-β-CD, inclusion complex, antitumor, cytotoxic

## Abstract

Glaucocalyxin A (GLA), is a diterpenoid extracted from Hara and has been studied for decades for its diverse bioactivities. However, GLA presents poor solubility in water and low bioavailability through oral administration which has hindered its application in the clinic. So in this study, we prepared the inclusion complex of GLA in SBE-β-CD by ultrasound method and evaluated its antitumor effect and cytotoxic effect on cancer cells. The production of GLA-SBE-β-CD inclusion complex was optimized using Box-Behnken design. The inhibitory effects of GLA and GLA-SBE-β-CD were investigated on the Hela, A549, HepG2, and SiHa cells *in vitro* by MTT staining assay. Pharmacokinetic studies were conducted on Sprague-Dawley mice via caudal injection to study the distribution, metabolism, and elimination of GLA-SBE-β-CD *in vivo*. Tumor-bearing nude mice were taken as the model and adopted to evaluate the inhibitory rate of GLA and GLA-SBE-β-CD on the transplanted tumor. A series of physical characterization results confirmed the fact that GLA-SBE-β-CD inclusion complex was successfully prepared. A production of 87.28% was achieved based on the Box-Behnken design. In the cancer cell inhibition studies, GLA and GLA-SBE-β-CD exhibited apparent concentration-dependent inhibitory actions on four kinds of tumor cells and better inhibition was achieved in GLA-SBE-β-CD group. The pharmacokinetic results showed that the duration of GLA in blood was prolonged and enhanced bioavailability was achieved. GLA and GLA-SBE-β-CD both showed an effective inhibition on the transplanted tumor growth, while the anti-tumor effect of GLA-SBE-β-CD (inhibitory rate of 45.80%) was significantly stronger than that of GLA (30.76%) based on the change of tumor weight and tumor volume.

## Introduction

Hara, a traditional Chinese herb widely grown in the south of China, has been used as anti-inflammatory, antipyretic, and stomachic agents in folk prescription since ancient times (Zhang et al., 1991; Li et al., 2000). As a famous diterpenoid extracted from the leaves of Hara (Li et al., 2000), GLA has been studied for decades about its diverse biological activities including antibacterial, antioxidative, anticoagulative, and DNA-protecting activities (Zhang et al., 1991; Liu et al., 2006). GLA also presents various levels of inhibition on cancer cells (Zhang et al., 2008; Gao et al., 2011; Tang et al., 2016) and tumor growth. Luckily, the mechanism of how it works was recently unraveled by Zhu et al., they found out that GLA inhibited five-zinc finger Glis (GLl 1) via regulating phosphatidylinositol 3 kinase/protein kinase B (P13K/Akt) signaling pathway thus induced apoptosis of these cancer cells (Zhu et al., 2018). Due to its hydrophobic structure, GLA has a poor aqueous solubility which has become a crucial obstacle in clinical application as a therapeutic agent. In order to improve the solubility of GLA and achieve higher bioavailability with lower toxicity *in vivo*, inclusion complexes with cyclodextrins are proposed as an effective method to solve these problems (Wang et al., 2011).

Cyclodextrins (CDs) are a family of crystalline, cyclic oligosaccharides consisting of α-1, 4-linked glucopyranose units. They have been popular as helpful molecular chelating agents in foods, pharmaceuticals, cosmetics these years (Arima et al., 2017; Sivakumar et al., 2018). β-cyclodextrins (β-CD) are most frequently used among its huge family due to low cost, and their cavity size makes it possible to include most of the common guest compounds. However, β-CD shows poor aqueous solubility, strong hemolysis, and severe nephrotoxicity which have hindered its practical application (Luke et al., 2010; Hafner et al., 2010; Wang et al., 2011). So synthetically modified β-CDs such as hydroxypropyl-β-cyclodextrin (HP-β-CD) and sulfobutylether-β-cyclodextrin (SBE-β-CD) emerge as requires (Eid et al., 2011; Hu et al., 2012; Kadari et al., 2017). The modified β-CD appears to be more water-soluble and less toxic than unmodified β-CD. Possible reasons are as follows: (1) The substituted group like sulfonic acid group in SBE-β-CD changed the property of β-CD surface thus enhanced its aqueous solubility. (2) As the molecular weight of modified β-CD increases, the reabsorption of renal tubules decreases which results in lower nephrotoxicity. And it is generally acknowledged that SBE-β-CD exhibits better performance than HP-β-CD in water solubility, toxicity, and solubility enhancement. In the research conducted by Shang et al., they prepared the inclusion complex of GLA with HP-β-CD, and the result showed 13-fold increase in aqueous solubility after the inclusion (Shang et al., 2011).

Hence, in this study, we prepared the inclusion complex of GLA in SBE-β-CD and the experimental conditions were optimized to obtain the highest inclusion efficiency. In consideration of the urgent need for drugs in cancer treatment field (Chen et al., 2016; Desantis et al., 2016), the antitumor effect of GLA-SBE-β-CD was investigated. The cytotoxicity of GLA-SBE-β-CD *in vitro* was evaluated on four cancer cells using MTT assay. The pharmacokinetic study was also conducted to analyze the pharmacokinetic characteristics of GLA-SBE-β-CD *in vivo*. To investigate the anti-tumor effect *in vivo*, a sarcoma 180 (S180) tumor-bearing nude mice model was established and the tumor growth was monitored before each dosing (Zhang et al., 2015). The inhibitory rate on S180 tumor was evaluated and compared with that of pure drug.

## Materials and methods

### Materials

#### Chemicals and reagents

GLA was kindly offered by the college of pharmaceutical science, Soochow University (Jiangsu, China). 5-fluorouracil was purchased from TCI Shanghai Co., Ltd (Shanghai, China). SBE-β-CD (Mw = 2.24 kD, mean substitution degree of 7) from Nanjing JuHuan Medical Technology Co., Ltd and bovine serum from ShangHai Luoshen Biotechnology Co., Ltd. Tryptose was obtained from Beyotime Biotechnology Co., Ltd. Methyl tert-butyl ether was purchased from Shanghai LingFeng Chemical Reagent Co., Ltd (Shanghai, China). Rubescensin was obtained from the National Institutes for Food and Drug Control. 3-(4, 5-dimethyl-2-thiazolyl)-2, 5-diphenyl-2-H-tetrazolium bromide was from Sigma-Aldrich (Saint Louis, MO). Methanol was of HPLC grade and the water being used in this experiment was ultrapure. Unless otherwise stated, all other materials were of analytical grade.

#### Cell lines and cell culture

The cell strains of human cervical cancer cell Hela, human lung carcinoma cell A549, human hepatoma cell HepG2, and human squamous cervical carcinoma cell SiHa were all obtained from Shanghai Cell Institution. The strains of the four cancer cells were conventionally cultured in RPMI-1640 medium supplemented with 10% fetal bovine serum in a weak alkaline condition (pH: 7.2–7.4). These cells were put under the condition of 37 °C, 5% carbon dioxide purge until used (Zhang et al., 2018).

#### Chromatographic conditions

A Shimazu HPLC equipped with a LC-20A pump and a PDA detector was used for the analysis of the concentration of GLA. The detection process was performed on a Kromasil reversed-phase C18 column (4.6 mm × 150 mm, 5 μm). The mobile phase is consisting of distilled water/methanol (1:1; v/v), and its flow rate was determined at 1.0 ml/min for isocratic elution. Column temperature was set at 25 °C. Detective wavelength was set at 231 nm based on the previous research by Zhang et al. (Zhang et al., 1991).

### Preparation and solubility studies of the inclusion compound

#### Preparation of GLA-SBE-β-CD inclusion complex

Ultrasound method and freeze-dried method were used in the preparation of GLA-SBE-β-CD inclusion complex. Aqueous solution of a specific amount of SBE-β-CD (m/v) was prepared in flasks in an ultrasound machine at a constant temperature, followed by increasing quantities of GLA (molar ratio of GLA to SBE-β-CD ranging from 1:5 to 1:1) in acetone slowly added dropwise into separate flasks. The resulting solutions were allowed to complex under ultrasound for some time until equilibrium and then placed in ambient temperature for 2 hours to cool down. Afterwards, the cooled solutions were freeze-dried in a vacuum desiccator to obtain the solid complex after being filtered through a 0.45 μm membrane (Hu et al., 2012; Kulkarni & Belgamwar, 2017).

The obtained inclusion complex was selected by measuring their inclusion efficiency: Specific amount of GLA-SBE-β-CD was accurately weighed and dissolved in the mobile phase, and the resulting solution of about 20 μl was injected into HPLC to analyze the concentration of GLA. As shown from the data in Supplementary Table S1, inclusion efficiency increased with the addition of SBE-β-CD and was over 80% when the ratio of GLA to SBE-β-CD reached 1:3. The inclusion efficiency exhibited no significant increase when the ratio continued to increase from 1:3. Considering the waste of the excipients, API and increasing difficulty of processing, a ratio of GLA to SBE 1:3 was determined as the optimal ratio of the inclusion complex.

#### Optimization of the condition in preparation of GLA- SBE-β-CD by Box–Behnken design (BBD)

Based on the above study, a molar ratio of GLA to SBE-β-CD 1:3 was determined as the optimal composition in the preparation of the inclusion complex. However, some other experimental conditions such as temperature, time, and concentration of the SBE-β-CD aqueous solution were directly related to the inclusion efficiency. So single factor tests of the 3 factors were performed and the general levels of each factor were determined (Supplementary Table S2). Then statistical approaches such as response surface method (RSM) was employed to maximize the production of the inclusion complex by optimizing operational factors including temperature (*A*), concentration of SBE-β-CD (*B*) and time (*C*). The response variable was inclusion efficiency (*Y*). In this paper, we adopted the BBD to perform the fitting. Based on the experimental design results, the mathematical model was established and the corresponding polynomial equations were generated and shown in Equation (1) below.

(1) $$Y=\beta_{0}+\beta_{1}A+\beta_{2}B+\beta_{3}C+\beta_{4}AB＋\beta_{5}AC+\beta_{6}BC+\beta_{7}A^{2}+\beta_{8}B^{2}+\beta_{9}C^{2}$$

Where *Y* is the response variable; β_0_ is intercept that represents the arithmetic average of all quantitative outcomes of 17 randomized experiments; β_1_–β_9_ are coefficients computed from the observed experimental values of *Y*; *A*, *B* and *C* are experimental factors; *AB*, *AC*, *BC* are factor interactions; *A^2^*, *B^2^*, and *C^2^* are quadratic terms.

#### Investigation of solubilization effect of GLA-SBE-β-CD

Excessive GLA and GLA-SBE-β-CD were separately added into 10 ml of distilled water and then were put in a thermostatic oscillator (25 °C) for 24 h. Afterwards, the obtained mixture was filtrated through 0.45 μm membrane and analyzed using HPLC method (Nair et al., 2014; Loh et al., 2016; Pan et al., 2017).

### Characterization

The inclusion complex of GLA with SBE-β-CD was investigated through the following methods (Bulani et al., 2016; Kulkarni & Belgamwar, 2017). GLA, SBE-β-CD and their physical mixture (GLA: SBE-β-CD = 1:3, molar ratio) were also characterized for comparison in order to determine whether the inclusion complex was formed.

### Differential scanning calorimetry

Thermal analysis of GLA, SBE-β-CD, their physical mixture and GLA-SBE-β-CD were conducted on a Phoenix DSC-204 (Phoenix Corporation, Selb, Germany) under a stream of nitrogen purge. Three to five milligrams samples were put on the crucibles and heated from 50–350 °C at a heating rate of 10 °C/min.

### Powder X-Ray diffraction

The powder X-Ray diffraction patterns of GLA, SBE-β-CD, their physical mixture and GLA- SBE-β-CD were investigated on a PW 1720 X-Ray generator and a PW 1710 diffractometer control (Philips Electronic Instruments, Mount Vernon, NY). The machine operated with a voltage and a current of 40 mA produced by Cu Kα radiation. Powders of GLA, SBE-β-CD, their physical mixture and GLA- SBE-β-CD were scanned from 2° to 35° at a scan step of 0.04°, and a scan speed of 0.02°/s. Jade5XRD pattern processing software (Materials Data, Inc, Irvine, CA) was used to analyze the X-Ray patterns of the samples.

### Scanning electron microscope analysis

The surface morphology of GLA, SBE-β-CD, and GLA-SBE-β-CD was evaluated with a scanning electron microscope (S-4800, Hitachi, Tokyo, Japan). The powder sample was adhered to the specimen stage using a dual adhesive tape, then was coated with an electrically conductive platinum film in a Hitachi Ion Sputter machine (E-1030, Hitachi, Japan). Afterwards, the prepared samples were observed using S-4800 Hitachi microscope and the photos of them were recorded.

### Study of the inhibition effect on human cancer cells: A549, Hela, HepG2, SiHa

Different concentrations of GLA and GLA-SBE-β-CD (1, 10, 100 μg/ml) were prepared using gradient dilution method with culture medium (RPMI-1640 medium with 10% fetal bovine serum).

The cell suspension of the four cancer cells at logarithmic growth were seeded into the 96-plate well (cell density was adjusted to 1 × 10^4^ cells per well) and exposed to different concentration of GLA or GLA-SBE-β-CD. The blank group was pure culture medium. The control group was given no drug but culture medium. The cells would be cultured for 24 h at the same condition as before (37 °C, 5%CO_2_). Twenty-four hours later, the RPMI-1640 medium was replaced by 10 μl MTT (3-[4,5-dimethylthiazol-2-yl]-2, 5-diphenyltetrazolium bromide) and 90 μl fresh culture medium and the cells would be cultured for another 4 h. After the incubation, 100 μl DMSO was added into each well and allowed to complex in a shaker for 10 min until complete dissolution of the formazan crystallization. StatFAx-2100 ELISA Reader was then used to detect the absorbance of the DMSO-cells mixed solution at the wavelength of 490 nm, and the detection was parallelly conducted in triplicate, the average value was used in the calculation of cell inhibitory rate.

The inhibitory rate was calculated using the following equation, Equation (2):

(2) $$\text{IR}=(1-({\text{OD}}_{\text{drug}}-{\text{OD}}_{\text{Blank}}))/({\text{OD}}_{\text{Control}}-{\text{OD}}_{\text{Blank}}))\times 100\%$$

where IR means the inhibition rate on cancer cells, OD_drug_ means the optical density of the drug group, the OD_blank_ means the optical density of the blank group, and the OD_Control_ means the optical density of the control group.

### Pharmacokinetic study

Drug levels in the blood after intravenous injection were investigated based on the LC/MS/MS method by Ren et al. (Ren et al., 2013).

12 Sprague-Dawley rats (weighed about 250 ± 30 g) were randomly divided into 2 groups: GLA group and GLA-SBE-β-CD group. Both of the 2 groups of rats were given no food but saline water for 12 hours before the experiment. After being weighed and numbered, the rats were injected separate drug through caudal vein at a dosage of 15 mg/kg body weight. Blood samples of about 0.3–0.4 mlwere collected from mice orbit at 0 min, 5 min, 10 min, 30 min, 45 min, 1 h, 2 h, 4 h, 6 h, 8 h, 12 h into heparinized centrifuged tubes. The blood samples were then centrifuged at the speed of 3000 r/min for 5 min at 4 °C to separate the plasma. The centrifuged plasma was stored at −20 °C in a refrigerator for sequent use.

The frozen samples were put into a 37 °C water bath to unfreeze. In order to make sure the concentration of GLA was in the linear range, the plasma samples were diluted with blank rat plasma. Ten microliters of rubescensin as an internal standard was mixed with 100 μl of plasma sample. The mixture was vortex extracted with 1 ml methyl tert-butyl ether (MTBE) for 5 min and centrifuged at 20,000 r/min, 4 °C for 10 min. Eight-hundred microliters of the supernatant in a 1.5 ml centrifuge tube was dried in a nitrogen blowing apparatus. Then the residue was dissolved in 100 μl mobile phase and injected 10 μl in LC/MS/MS to analyze the content of GLA.

### Inhibition of GLA and its inclusion complex on the growth of S180 tumor in tumor-bearing nude mice

The nude mice used in this experiment (25–35 g, male and female) were provided by the Experimental Animal Center of Nanjing University of Chinese Medicine (NO.SYXK Su 2002-0123). S180 cells were from Jiangsu Provincial Animal Inspection Station and were kept in our laboratory by subculture.

The anti-tumor effect experiment described here was under the approval of Nanjing Medical University Institutional Animal Care and Use Committee and the ‘Principle of Laboratory Animal Care’ was strictly adhered during this experiment.

S180 cells at a logarithmic period were selected as the target samples in this experiment, the concentration of these cells was adjusted to 1 × 10^7^/ml using DMEM medium (without bovine serum). Then 0.2 ml of S180 cell suspension was inoculated hypodermically into the right forelimbs of a group of nude mice. After 2 weeks, the mice with well-grown tumors were put to death by cervical vertebra dislocation to separate the tumor under aseptic conditions. The weights of these tumors were accurately recorded. The obtained tumors were then put into a glass tissue homogenizer to be ground. The homogenate of the well-grown tumors was added to sterile vessels and diluted into cell suspension with a specific amount of saline (homogenate: saline = 1:3, v/v). A new group of the nude mice was inoculated with 0.2 ml cell suspension of the homogenate, 10 days later 40 of them with well-grown tumors (diameter about 1 cm) were selected as the samples in the anti-tumor research (Zhang et al., 2015). Forty of the selected mice were randomly divided into 4 groups: Model group, 5-fluorouracil group (5-FU group), GLA group, and GLA-SBE-β-CD group. With the mice of Model group being given the equal volume of saline, corresponding drugs were intravenously injected into other three groups of mice through a caudal vein at 1, 3, 5, 7, 9 days, since the first injection (Zhang et al., 2015). The dosage of the drugs was related to the body weight which was 15 mg/Kg body weight. The longest diameter of the tumor (D_max_), the corresponding short diameter (D_min_) which was perpendicular to the longest one, weight of the mice would be recorded before each injection. The recorded D_max_ and D_min_ would be used to calculate the volume of the tumor according to the following equation (Equation (3)).

(3) $$V_{\text{tumor}}=1/2\times D_{\text{max}}\times {\left(D_{\text{min}}\right)}^{2}$$

where V_tumor_ means the volume of the tumor of each group, D_max_ and D_min_ stand for the longest diameter and the corresponding short diameter, respectively.

On the 11th day, all the experimental mice would be put to death and the tumors inside them, separated and weighed, D_max_ and D_min_ were recorded.

## Results and discussions

### Optimization of the condition in preparation of GLA- SBE-β-CD by BBD

The actual fitting equation was shown in Equation (4) below:

(4) $$Y=-173.65+9.42A+2.34B+0.974C+9.89\times 10^{-3}AB-2.687\times 10^{-3}AC-3.67\times 10^{-3}BC-0.139A^{2}-0.0685B^{2}-0.011C^{2}$$

**Figure 1.**
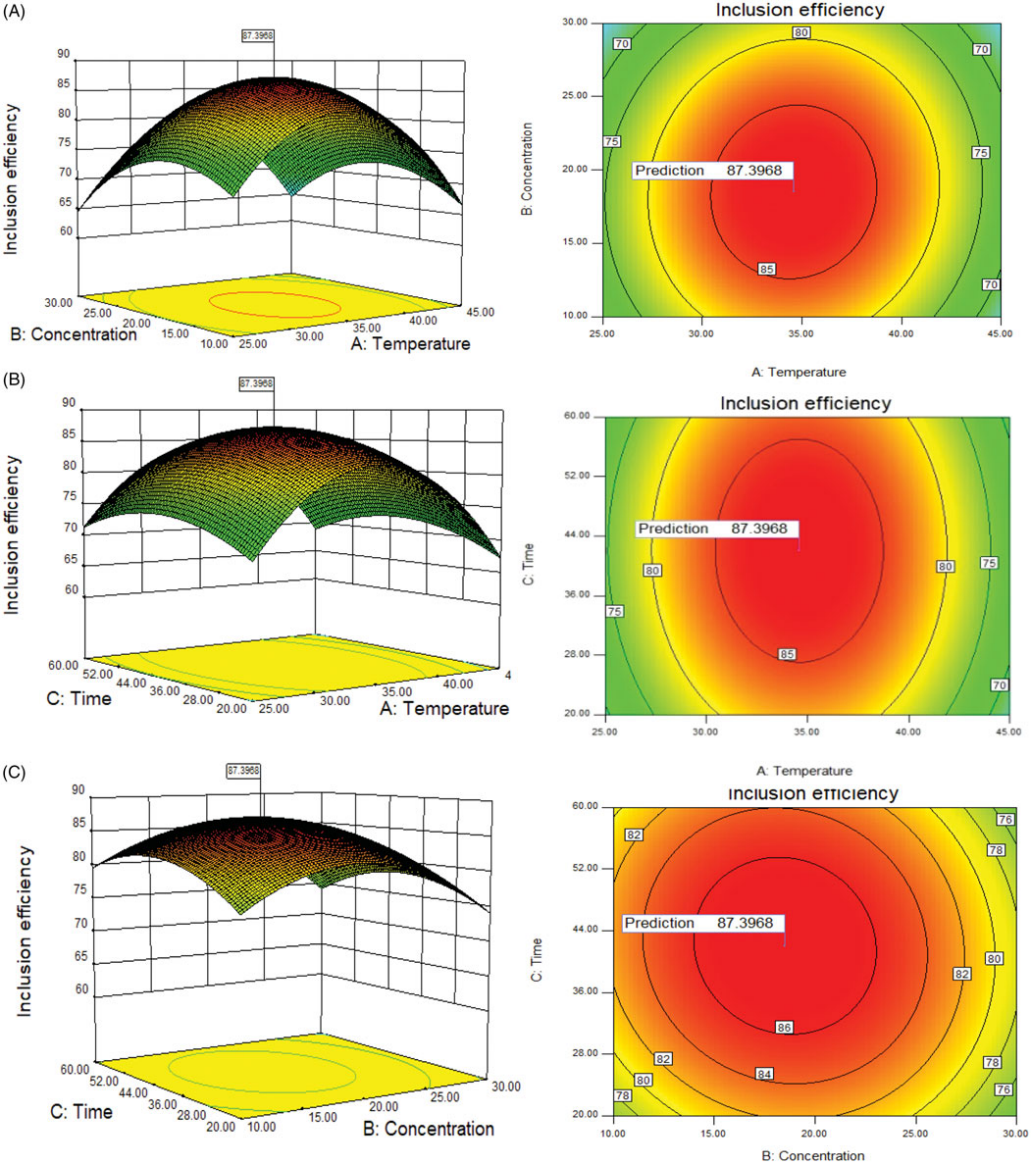
(A) Response surface graph (3D) and contour graph showing the effect of the temperature (*A*) and concentration of SBE-β-CD *(B)*, (B) the temperature (*A*) and inclusion time *(C)*, (C) the concentration of SBE-β-CD (*B*) and inclusion time *(C)* added on the response (inclusion efficiency).

BBD matrix and the response values were displayed in Supplementary Table S3. The 3 D respond surfaces and contour plots were presented in Figure 1. Analysis of variance (ANOVA) for the response surface quadratic model was exhibited in Supplementary Table S4.

The results showed that the model has high *F* value (33.34) and extremely low *p* value (<.0001), which indicated that the model is statistically significant. The *p* value of ‘lack of fit’ was .0666 (>.05) indicated insignificant difference between the actual results and the fitting model. The *R*-squared and the Adj *R*-squared are 0.9772 and 0.9479, respectively, indicating a well correlation between the experimental factors and response variable. The *F* value of temperature (*A*), concentration of SBE-β-CD (*B*) and inclusion time (*C*) were 1.57, 5.86, 0.91, respectively, which indicates that the inclusion efficiency was influenced most by the concentration of SBE-β-CD (*B*) and least by inclusion time (*C*). Coefficient of variation (CV) value is 3.47% (<15%) which means the results were all in normal standard and well-concentrated.

The response surface graph can show the relationship between each variable and the inclusion efficiency: the steeper the surface is, the more sensitive the response variable is to the change of the variables. So we can directly tell that the concentration of SBE-β-CD (*B*) has the biggest influence on the inclusion efficiency. The shape of the contour map can clearly reflect whether or not the interaction between the two variables is significant or not: the elliptical contour line indicates that the interaction between the two factors is significant, on the contrary, the roundness of contour line indicates that the interaction between two factors is less significant. So we can see that the interactions between *AB*, *AC* are significant, while insignificant between *BC*.

The optimum experimental parameters and theoretical maximum inclusion efficiency can be obtained according to the calculation results using Design expert software from Stat-Ease, Inc.（MN 55413, USA). Considering the actual test conditions, the SBE-β-CD concentration of 18.5%, inclusion temperature of 35 °C and inclusion time of 42 min were selected. The preparation was conducted under the optimum condition, a mean production of 87.28%, relative standard deviation (RSD) of 1.02% was achieved. This production was a little lower than the theoretical value (87.40%) but within the error range.

### Investigation of solubilization effect of GLA-SBE-β-CD

The saturated solubility of GLA was 0.213 mg/ml, while the value of GLA-SBE-β-CD was 17.96 mg/ml which was 84.3 times that of GLA indicating that the GLA-SBE-β-CD complex had largely improved the solubility of GLA in water.

## Characterization

The formation of GLA-SBE-β-CD inclusion complex was confirmed by the results of DSC, FTIR, PXRD, and SEM.

### Differential scanning calorimetry

**Figure 2.**
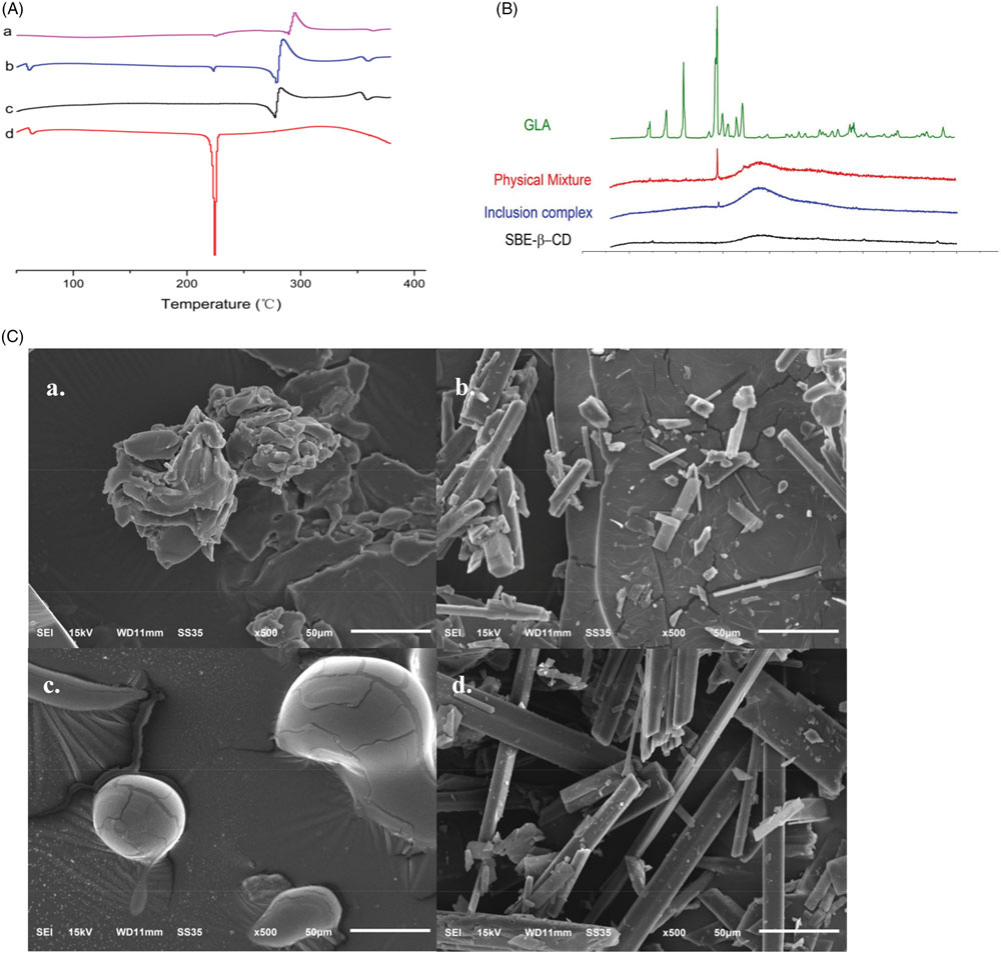
(A) DSC thermograms of GLA-SBE-β-CD inclusion complex (a), their physical mixture (b), SBE-β-CD (c) and GLA (d). (B) PXRD patterns of GLA, their physical mixture, GLA- SBE-β-CD inclusion complex and SBE-β-CD. (C). SEM photo of GLA- SBE-β-CD inclusion complex (a), their physical mixture (b), SBE-β-CD (c), and GLA (d).

Thermal behaviors of GLA-SBE-β-CD inclusion complex (a) and their physical mixture (b) were studied by DSC and displayed in Figure 2(A). The differential scanning calorimetry (DSC) curves of GLA (c) and SBE-β-CD (d) were also recorded for comparison in order to verify the inclusion or partial inclusion of GLA in SBE-β-CDs cavities. The curve of GLA showed a sharp endothermic peak at 225 °C corresponding to its melting point. An irregular peak of SBE-β-CD emerged at 300 °C resulting from the degradation process. Compared to pure GLA and SBE-β-CD, the endothermic peaks of GLA at 225 °C in the physical mixture were weakened and the peaks of SBE-β-CD were intensified. This result may attribute to some molecular interactions between GLA and SBE-β-CD. The thermogram of GLA-SBE-β-CD showed that the original endothermic peak of pure GLA has disappeared which indicated the complete inclusion of GLA in SBE-β-CD.

### Powder X-Ray diffraction

PXRD patterns of pure GLA, SBE-β-CD, GLA-SBE-β-CD inclusion complex and their physical mixture were displayed in Figure 2(B). GLA pattern exhibited multiple sharp and well-defined peaks, indicating that GLA exists in a typical crystalline pattern. On the contrary, SBE-β-CD exhibited a flat curve with only one weak ascent in the scanning range demonstrating its amorphous state. All major characteristic peaks of GLA were observed in the diffractograms of their physical mixture but with lower intensity, which could attribute to the reduction of the particle size during physical mixing or some interactions between GLA and SBE-β-CD. As we can see from the GLA-SBE-β-CD pattern, most of the characteristic crystalline peaks of GLA had disappeared. This phenomenon suggested that GLA was no more in crystalline form and was included in SBE-β-CD cavity. PXRD results were in agreement with previous studies that GLA has been included in SBE-β-CD.

### Scanning electron microscopy analysis

The scanning electron method analysis (SEM) photo of GLA, SBE-β-CD, their physical mixture, and GLA-SBE-β-CD were displayed in Figure 2(C). As we can see from the picture, SBE-β-CD showed a spherical shape with a smooth boundary while GLA had sharp angles and was in a smooth columnar crystal shape. In the photo of their physical mixture, the spherical shape of SBE-β-CD and columnar crystal of GLA were obviously observed. However, the specific morphological characteristics of SBE-β-CD and GLA no longer existed in the photo of GLA-SBE-β-CD. The particle of GLA-SBE-β-CD was in an amorphous state and was a bit bigger than GLA and SBE-β-CD. From Figure 2(C), we can find that the surface morphology of GLA-SBE-β-CD was obviously different from those of GLA, SBE-β-CD, the physical mixture, and a new material was formed.

### Inhibition of GLA and its inclusion complex on human cancer cells

**Figure 3.**
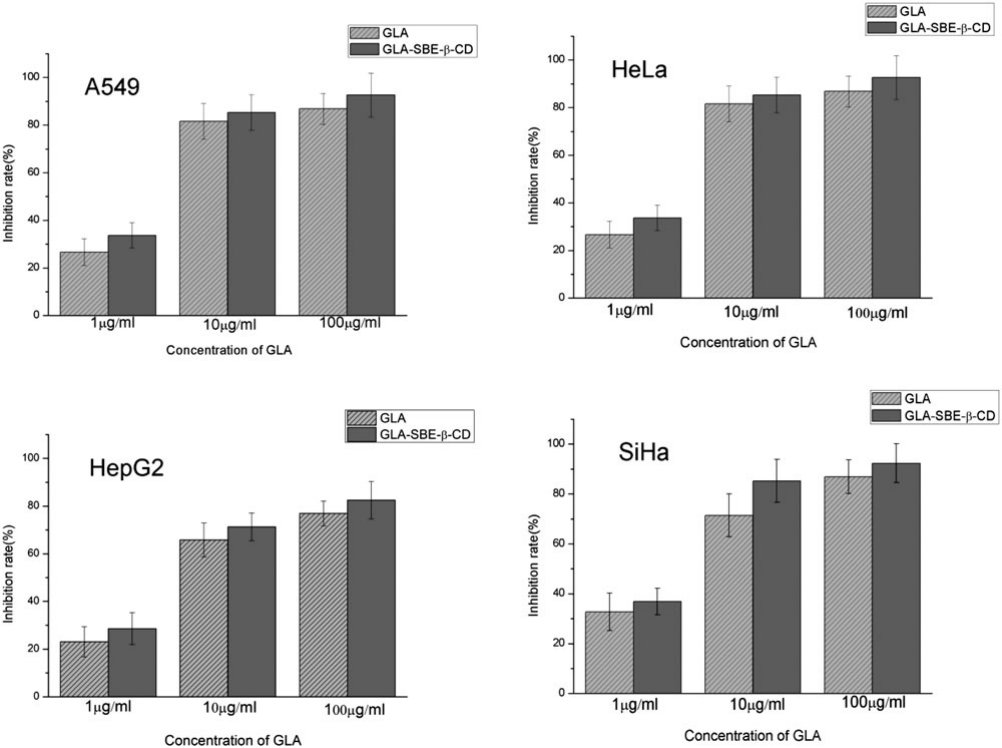
The inhibition rate of GLA and GLA-SBE-β-CD on four human cancer cells: A549, HeLa, HepG2, and SiHa.

The inhibitory rate of GLA and GLA-SBE-β-CD on A549, Hela, HepG2, SiHa cells at different concentrations were displayed in Figure 3. Both of GLA and its inclusion complex had significant inhibition on these four tumor cells while it should be noticed that GLA-SBE-β-CD showed greater inhibition comparing to pure GLA. For GLA-SBE-β-CD, when the concentration increases to 100 μg/ml, the inhibitory rate on the four tumor cells were all over 80% and best inhibition (>90%) were achieved on A549 and SiHa cells.

### Pharmacokinetic study

**Figure 4.**
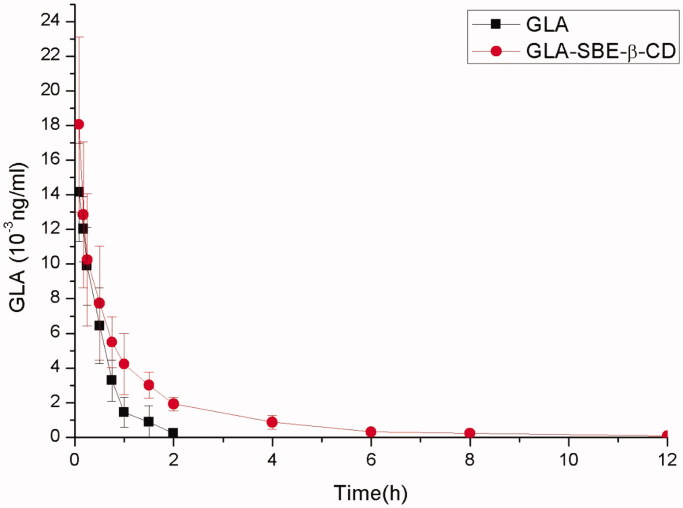
The curve of the mean plasma drug concentration versus time in rats after intravenous injection of GLA-SBE-β-CD or GLA.

Pharmacokinetic studies were conducted to study the distribution, metabolism, and elimination of GLA-SBE-β-CD *in vivo* (Uchida et al., 2015). GLA and GLA-SBE-β-CD were injected into the Sprague-Dawley (SD) rats through caudal vein, the concentration of GLA in plasma at different timepoints was detected and depicted in Figure 4.

Pharmacokinetic parameters were calculated based on the concentration-time profiles using DAS2.0 software (Mathematical Pharmacology Professional committee of China, Shanghai, China). The parameters were shown in Supplementary Table S5. As the data showed, the clearance rate of GLA-SBE-β-CD and GLA *in vivo* was 0.909 ± 0.237, 1.339 ± 0.386 L/kg/h, respectively. The clearance of GLA-SBE-β-CD was 2/3 that of pure GLA which indicates that the clearance rate of GLA was slowed down after being prepared into inclusion compound. And the area under the curve (AUC) of GLA-SBE-β-CD (17.565 ± 5.063 μg·h/ml) was twice greater than that of GLA (8.079 ± 2.600 μg·h/ml) indicating greater bioavailability *in vivo* with the injection of the inclusion compound. The *t*_1/2_ and MRT (mean retaining time) of the inclusion was apparently longer than pure drug. According to the data mentioned above, the conclusion could be made that, the bioavailability of GLA-SBE-β-CD was greater than GLA and inclusion complex has prolonged the duration of GLA in blood and in tissue. This phenomenon indicated that the inclusion complex possessed sustained release effects *in vivo* and may due to the delay of the release from the SBE-β-CD cavity.

### Inhibition of GLA and its inclusion complex on the growth of transplanted S180 tumors

**Figure 5.**
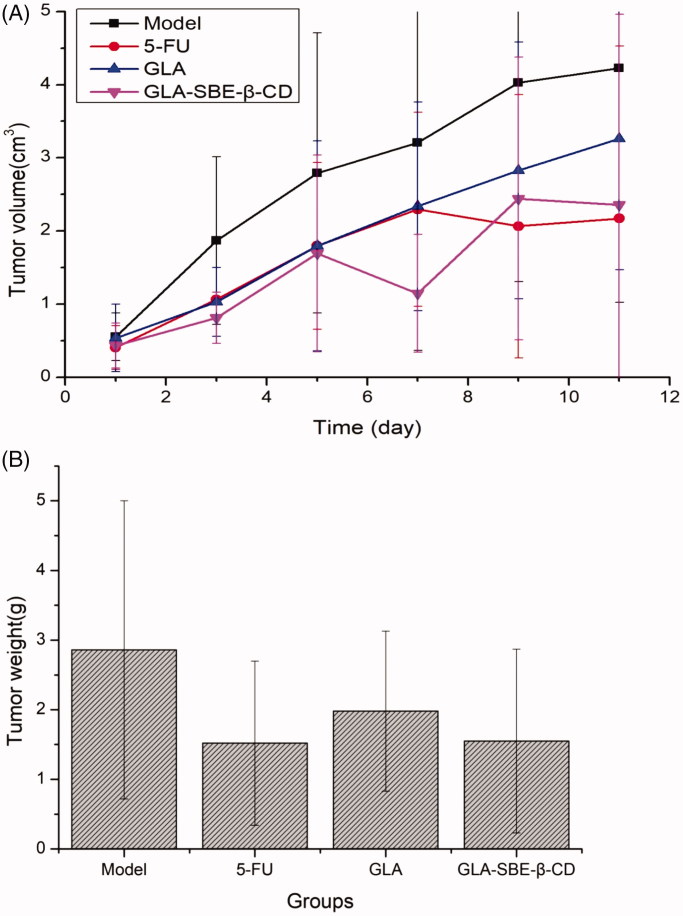
(A) Tumor volume change after intravenous injection of GLA and GLA-SBE-β-CD. (B) The tumor weight at day 11 after the first injection.

The inhibition efficiency of GLA and its inclusion complex on tumor growth were assessed with respect to tumor weight, tumor volume, and body weight of S180 tumor-bearing mice. The volume change of the tumors was calculated using Equation 5 and were shown in Figure 5(A). The tumors were separated at 11th day and the tumor weight was recorded. The mean tumor weight was depicted in Figure 5(B).

From Figure 5(A) we can see, 5-FU, GLA, and its inclusion complex exhibited different levels of suppression on the tumor growth based on the fact that the volume of S180 tumor was always smaller than Model group since the first injection. The tumor volume of GLA group and the Model group, both showed an increasing trend during the experiment while GLA group displayed a gentle growth and ended up with a smaller volume than the Model group. Greater inhibition was achieved by GLA-SBE-β-CD and 5-FU group for which the tumor growth was significantly inhibited according to the volume reduction on the 5th or 7th day of the injection.

According to Figure 5(B), GLA group presented a lighter mean tumor weight than the Model group, while GLA-SBE-β-CD group showed almost 50% reduction in mean tumor weight compared to Model group, which demonstrated remarkable inhibition of GLA-SBE-β-CD on tumor growth. The inhibition efficiency was calculated using the following equation (Equation 5).

(5) $$IR=\frac{W_{\text{model}}-W_{\text{drug}}}{W_{\text{model}}}\times 100\%$$

where IR means the inhibitory rate, W_drug_ and W_model_ stand for mean tumor weight of the drug group and the Model group, respectively.

From the results we can see, the greatest but similar inhibitions were obtained by GLA-SBE-β-CD and 5-FU group with the inhibition efficacy of 45.80%, 46.85%, respectively, while the value of GLA group is 30.76% which is notably inferior to the other 2 groups. It can be concluded that the anti-tumor activity of GLA *in vivo* has significantly enhanced after its inclusion. This result may due to sustained release of the inclusion complex thus extended the duration *in vivo* and enhanced the drug effect.

### Data analysis

DAS 2.0 (Shanghai, China) was used in the analysis of the data. Statistics were presented as $\bar{x}\pm s$. One-Way analysis of variance (ANOVA) was used in the comparison of multi-group variables. Significant difference was regarded as *p* < .05.

## Conclusion

GLA was a diterpenoid with poor absorption and low bioavailability leading to poor antitumor effect. In this paper, we introduced GLA-SBE-β-CD inclusion complex injection and the antitumor effect, and inhibition effect on cancer cells were enhanced. Pharmacokinetic study on SD rats showed that inclusion complex had prolonged the duration of GLA in plasma. As the anti-tumor study and cytotoxic experiments exhibited, GLA and its inclusion complex with SBE-β-CD both had remarkable inhibition on the growth of S180 tumor in tumor-bearing mice and significant cytotoxicity, while the inhibition of GLA-SBE-β-CD on tumor growth and cancer cells were both obviously stronger than GLA.

## Supplementary Material

Supplementary_Figures_and_Tables.docx
