# Supplementary material for: Preparation, optimization of the inclusion complex of glaucocalyxin A with sulfobutylether-β-cyclodextrin and antitumor study
Source: Drug Deliv. 2019 Mar 21;26(1):309–17. doi: 10.1080/10717544.2019.1568623 (PMC6442205; doi:10.1080/10717544.2019.1568623)
Supplement: Supplementary_Figures_and_Tables.docx [file IDRD_A_1568623_SM5238.docx]

Table S1. Entrapment efficiency of GLA in SBE-β-CD in different molar ratio

| Number | Molar ratio of GLA/SBE-β-CD | Entrapment effiency % |
| --- | --- | --- |
| 1 | 1:1 | 64.91% |
| 2 | 1:2 | 72.77% |
| 3 | 1:3 | 81.23% |
| 4 | 1:4 | 83.76% |
| 5 | 1:5 | 84.45% |

Table S2. Factors and levels of Box-Benhnken test

| Factor  Level | A | B | C |
| --- | --- | --- | --- |
|  | Temperature(°C) | Concentration of SBE-β-CD(M/V) | Inclusion time(min) |
| -1 | 30℃ | 10% | 20 |
| 0 | 40℃ | 20% | 40 |
| 1 | 50℃ | 30% | 60 |

Table S3 Box-Benhnken design matrix and response values

| Run | A | B | C | R |
| --- | --- | --- | --- | --- |
| 1 | 1 | 0 | 0 | 72.32 |
| 2 | -1 | 0 | 0 | 76.19 |
| 3 | 0 | 0 | 0 | 86.79 |
| 4 | 0 | 0 | 0 | 85.85 |
| 5 | 0 | 0 | 0 | 85.25 |
| 6 | 0 | 0 | -1 | 84.28 |
| 7 | 1 | -1 | 1 | 65.53 |
| 8 | -1 | -1 | -1 | 65.45 |
| 9 | 1 | 1 | 1 | 58.92 |
| 10 | 1 | 1 | -1 | 60.21 |
| 11 | 0 | -1 | 0 | 80.32 |
| 12 | -1 | 1 | -1 | 57.82 |
| 13 | -1 | 1 | 1 | 60.41 |
| 14 | 1 | -1 | -1 | 60.22 |
| 15 | -1 | -1 | 1 | 67.31 |
| 16 | 0 | 0 | 1 | 83.42 |
| 17 | 0 | 1 | 0 | 82.21 |

TableS4. Analysis of variance（ANOVA）for the response surface quadratic model

| Index | Value | Index | Value |
| --- | --- | --- | --- |
| Std.Dev | 2.52 | R-Squared | 0.9972 |
| Mean | 72.50 | Adj R-Squared | 0.9479 |
| C.V.% | 3.47 | Pred R-Squared | 0.7868 |
| Press | 414.69 | Adeq Precisior | 13.975 |

Table S5.The pharmacokinetic parameters

| Parameters | Unit | GLA injection | GLA-SBE-β-CD |
| --- | --- | --- | --- |
| AUC_0~τ_ | μg•h•mL^-1^ | 8.006$\pm$2.549 | 17.279$\pm$4.899 |
| AUC_0~∞_ | μg•h•mL^-1^ | 8.079$\pm$2.600 | 17.565$\pm$5.063 |
| MRT | h | 0.409$\pm$0.076 | 1.684$\pm$0.351 |
| t_1/2_ | h | 0.28$\pm$0.047 | 2.191$\pm$0.892 |
| V | L/Kg | 0.536$\pm$0.171 | 2.737$\pm$1.101 |
| CLz | L/(kg•h) | 1.339$\pm$0.386 | 0.909$\pm$0.237 |
| T_max_ | h | 0.095$\pm$0.037 | 0.08 |
| C_max_ | μg•mL^-1^ | 14.287$\pm$3.11 | 18.06$\pm$5.532 |
